# 1,1-Dibenzyl-3-(4-fluoro­benzo­yl)thio­urea

**DOI:** 10.1107/S1600536811026687

**Published:** 2011-07-09

**Authors:** Mohd Faizal Md Nasir, Ibrahim N. Hassan, Wan Ramli Wan Daud, Bohari M. Yamin, Mohammad B. Kassim

**Affiliations:** a Fuel Cell Institute, Universiti Kebangsaan Malaysia, UKM 43600 Bangi Selangor, Malaysia; b Department of Chemical and Process Engineering, Faculty of Engineering, Universiti Kebangsaan Malaysia, UKM 43600 Bangi Selangor, Malaysia; c School of Chemical Sciences and Food Technology, Faculty of Science and Technology, Universiti Kebangsaan Malaysia, UKM 43600 Bangi Selangor, Malaysia

## Abstract

In the title compound, C_22_H_19_FN_2_OS, the 2-fluoro­benzoyl group adopts a *trans* conformation with respect to the thiono S atom across the N—C bond. In the crystal, inter­molecular N—H⋯S, C—H⋯S and C—H⋯O hydrogen bonds link the mol­ecules, forming a two-dimensional network parallel to (101).

## Related literature

For standard bond lengths, see: Allen *et al.* (1987 ▶). For related structures, see: Nasir *et al.* (2011 ▶); Yamin & Hassan (2004 ▶); Hassan *et al.* (2008*a* ▶,*b* ▶,*c* ▶, 2009 ▶). For the synthesis, see: Hassan *et al.* (2008*a* ▶).
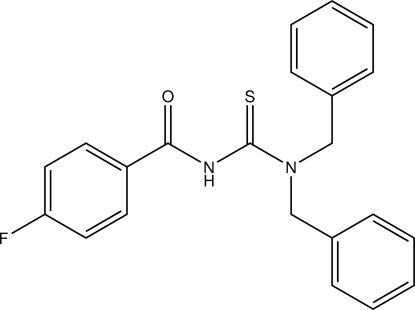


## Experimental

### 

#### Crystal data

C_22_H_19_FN_2_OS
*M* *_r_* = 378.45
Monoclinic, 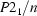

*a* = 10.683 (3) Å
*b* = 7.026 (2) Å
*c* = 26.435 (7) Å
β = 101.100 (6)°
*V* = 1946.9 (10) Å^3^
*Z* = 4
Mo *K*α radiation
μ = 0.19 mm^−1^
*T* = 298 K
0.42 × 0.21 × 0.18 mm

#### Data collection

Bruker SMART APEX CCD area-detector diffractometer
Absorption correction: multi-scan (*SADABS*; Sheldrick, 2000 ▶) *T*_min_ = 0.925, *T*_max_ = 0.967
8644 measured reflections
3425 independent reflections
2659 reflections with *I* > 2σ(*I*)
*R*_int_ = 0.027

#### Refinement

*R*[*F* ^2^ > 2σ(*F* ^2^)] = 0.059
*wR*(*F* ^2^) = 0.138
*S* = 1.15
3425 reflections
244 parameters
H-atom parameters constrained
Δρ_max_ = 0.25 e Å^−3^
Δρ_min_ = −0.23 e Å^−3^

### 

Data collection: *SMART* (Bruker, 2000 ▶); cell refinement: *SAINT* (Bruker, 2000 ▶); data reduction: *SAINT*; program(s) used to solve structure: *SHELXS97* (Sheldrick, 2008 ▶); program(s) used to refine structure: *SHELXL97* (Sheldrick, 2008 ▶); molecular graphics: *ORTEPIII* (Burnett & Johnson, 1996 ▶), *XP* in *SHELXTL* (Sheldrick, 2008 ▶) and *PLATON* (Spek, 2009 ▶); software used to prepare material for publication: *SHELXTL* and *PLATON*.

## Supplementary Material

Crystal structure: contains datablock(s) global, I. DOI: 10.1107/S1600536811026687/dn2702sup1.cif

Structure factors: contains datablock(s) I. DOI: 10.1107/S1600536811026687/dn2702Isup2.hkl

Supplementary material file. DOI: 10.1107/S1600536811026687/dn2702Isup3.cml

Additional supplementary materials:  crystallographic information; 3D view; checkCIF report

## Figures and Tables

**Table 1:** Hydrogen-bond geometry (Å, °)

*D*—H⋯*A*	*D*—H	H⋯*A*	*D*⋯*A*	*D*—H⋯*A*
N1—H1⋯S1^i^	0.86	2.61	3.422 (2)	159
C1—H1*A*⋯S1^i^	0.93	2.87	3.727 (3)	154
C4—H4⋯O1^ii^	0.93	2.50	3.322 (4)	147

Symmetry codes: (i) 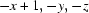; (ii) 
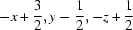
.

**Fig. 1.**
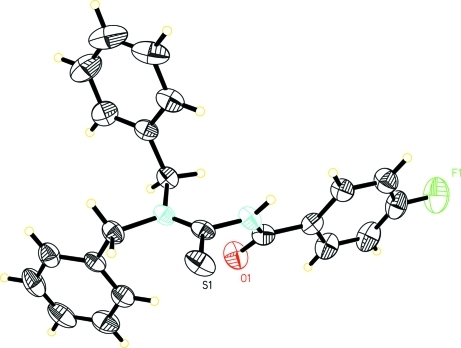
The molecular structure of (I), with the atoms labeling scheme and displacement ellipsoids are drawn at the 50% probability level. H atoms are represented as small spheres of arbitrary radii.

**Fig. 2.**
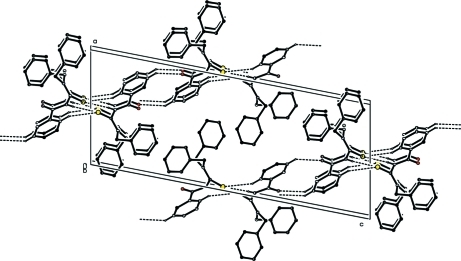
Partial packing of (I) view down the b axis. H bonds are shown as dashed lines. H atoms not involved in hydrogen bondings have been omitted for clarity.

## supplementary crystallographic information

### Comment

The title compound, I, is a thiourea derivative of dibenzylamine analogous to our previous reported, 1,1-sibenzyl-3-(3-chlorobenzoyl)thiourea, II (Nasir *et al.* 2011). The molecule maintains the the same *trans* and *cis* conformation for both the 3-fluorobenzoyl and the dibenzylamine groups, respectively, relative to the S atom across the N2—C8 bond (Fig 1). The dihedral angle between the phenyl ring, (C1—C6), and the thiourea fragment, (S1/N1/N2/C8) is 70.95 (13)° , whereas in II was 72.9 (2)°. The bond lengths and angles in the molecules are in normal ranges (Allen *et al.*, 1987) and comparable with those of II. However, the C=S bond length [1.678 (2)Å] is slightly longer than that of (II) [1.672 (6)Å]

Both phenyl rings, [C10/C11/C12/C13/C14/C15] and [C17/C18/C19/C20/C21/C22] are essentially planar and are twisted to each other by a dihedral angle of 22.4 (4)°. The intermolecular N1—H1···S1, C1—H1A···S and C4—H4···O1 hydrogen bonds (Table 1,) links the molecules into two dimensional ribbon parallel to the (1 0 1) plane (Fig 2).

### Experimental

The title compound was synthesized according to a previously reported compound (Hassan *et al.*, 2008*a*). A colourless crystal, suitable for X-ray crystallography, was obtained by a slow evaporation from ethanolic solution at room temperature (yield 87%).

### Refinement

H atoms of C and N atoms were positioned geometrically and allowed to ride on their parent atoms, with *U*_iso_= 1.2*U*_eq_ (C) for aromatic 0.93 Å, *U*_iso_ = 1.2*U*_eq_ (C) for CH_2_ 0.97 Å, *U*_iso_ = 1.2*U*_eq_ (N) for N—H 0.86 Å.

### Crystal data

**Table:**

C_22_H_19_FN_2_OS	*F*(000) = 792
*M**_r_* = 378.45	*D*_x_ = 1.291 Mg m^−^^3^
Monoclinic, *P*2_1_/*n*	Melting point: 410 K
Hall symbol: -P 2yn	Mo *K*α radiation, λ = 0.71073 Å
*a* = 10.683 (3) Å	Cell parameters from 2659 reflections
*b* = 7.026 (2) Å	θ = 2.0–25.0°
*c* = 26.435 (7) Å	µ = 0.19 mm^−^^1^
β = 101.100 (6)°	*T* = 298 K
*V* = 1946.9 (10) Å^3^	Block, colourless
*Z* = 4	0.42 × 0.21 × 0.18 mm

### Data collection

**Table:**

Bruker SMART APEX CCD area-detector diffractometer	3425 independent reflections
Radiation source: fine-focus sealed tube	2659 reflections with *I* > 2σ(*I*)
graphite	*R*_int_ = 0.027
ω scans	θ_max_ = 25.0°, θ_min_ = 2.0°
Absorption correction: multi-scan (*SADABS*; Sheldrick, 2000)	*h* = −12→8
*T*_min_ = 0.925, *T*_max_ = 0.967	*k* = −8→8
8644 measured reflections	*l* = −31→28

### Refinement

**Table:**

Refinement on *F*^2^	Primary atom site location: structure-invariant direct methods
Least-squares matrix: full	Secondary atom site location: difference Fourier map
*R*[*F*^2^ > 2σ(*F*^2^)] = 0.059	Hydrogen site location: inferred from neighbouring sites
*wR*(*F*^2^) = 0.138	H-atom parameters constrained
*S* = 1.15	*w* = 1/[σ^2^(*F*_o_^2^) + (0.0542*P*)^2^ + 0.5146*P*] where *P* = (*F*_o_^2^ + 2*F*_c_^2^)/3
3425 reflections	(Δ/σ)_max_ < 0.001
244 parameters	Δρ_max_ = 0.25 e Å^−^^3^
0 restraints	Δρ_min_ = −0.23 e Å^−^^3^

### Special details

**Table:**

Geometry. All esds (except the esd in the dihedral angle between two l.s. planes) are estimated using the full covariance matrix. The cell esds are taken into account individually in the estimation of esds in distances, angles and torsion angles; correlations between esds in cell parameters are only used when they are defined by crystal symmetry. An approximate (isotropic) treatment of cell esds is used for estimating esds involving l.s. planes.
Refinement. Refinement of F^2^ against ALL reflections. The weighted R-factor wR and goodness of fit S are based on F^2^, conventional R-factors R are based on F, with F set to zero for negative F^2^. The threshold expression of F^2^ > 2sigma(F^2^) is used only for calculating R-factors(gt) etc. and is not relevant to the choice of reflections for refinement. R-factors based on F^2^ are statistically about twice as large as those based on F, and R- factors based on ALL data will be even larger.

### Fractional atomic coordinates and isotropic or equivalent isotropic displacement parameters (Å^2^)

**Table:**

	*x*	*y*	*z*	*U*_iso_*/*U*_eq_	
F1	0.8992 (2)	−0.7024 (3)	0.17795 (9)	0.1137 (8)	
S1	0.47635 (8)	0.26603 (10)	0.02677 (3)	0.0641 (3)	
O1	0.5719 (2)	0.0400 (3)	0.17142 (7)	0.0736 (6)	
N1	0.5164 (2)	−0.0377 (3)	0.08698 (7)	0.0502 (6)	
H1	0.5210	−0.1222	0.0639	0.060*	
N2	0.3452 (2)	0.1514 (3)	0.09743 (7)	0.0453 (5)	
C1	0.6626 (3)	−0.3835 (4)	0.11002 (10)	0.0570 (7)	
H1A	0.6047	−0.3773	0.0789	0.068*	
C2	0.7407 (3)	−0.5402 (5)	0.12067 (12)	0.0697 (8)	
H2	0.7369	−0.6391	0.0970	0.084*	
C3	0.8236 (3)	−0.5463 (5)	0.16670 (13)	0.0742 (9)	
C4	0.8339 (3)	−0.4041 (6)	0.20216 (12)	0.0807 (10)	
H4	0.8924	−0.4120	0.2331	0.097*	
C5	0.7554 (3)	−0.2481 (5)	0.19116 (10)	0.0694 (9)	
H5	0.7605	−0.1501	0.2151	0.083*	
C6	0.6688 (2)	−0.2352 (4)	0.14481 (9)	0.0495 (6)	
C7	0.5840 (2)	−0.0665 (4)	0.13658 (9)	0.0507 (7)	
C8	0.4407 (3)	0.1238 (3)	0.07298 (8)	0.0460 (6)	
C9	0.2742 (3)	0.3312 (4)	0.09357 (10)	0.0532 (7)	
H9A	0.1842	0.3052	0.0815	0.064*	
H9B	0.3027	0.4123	0.0683	0.064*	
C10	0.2914 (3)	0.4349 (3)	0.14432 (9)	0.0482 (6)	
C11	0.4099 (3)	0.4550 (4)	0.17540 (11)	0.0627 (8)	
H11	0.4801	0.3968	0.1659	0.075*	
C12	0.4261 (4)	0.5594 (5)	0.22019 (12)	0.0768 (10)	
H12	0.5069	0.5718	0.2406	0.092*	
C13	0.3240 (4)	0.6450 (4)	0.23477 (12)	0.0796 (10)	
H13	0.3351	0.7164	0.2649	0.096*	
C14	0.2052 (4)	0.6251 (4)	0.20481 (13)	0.0776 (10)	
H14	0.1352	0.6823	0.2148	0.093*	
C15	0.1887 (3)	0.5196 (4)	0.15949 (11)	0.0625 (8)	
H15	0.1076	0.5062	0.1393	0.075*	
C16	0.2919 (3)	0.0015 (4)	0.12613 (9)	0.0506 (7)	
H16A	0.2790	0.0517	0.1589	0.061*	
H16B	0.3517	−0.1036	0.1330	0.061*	
C17	0.1667 (3)	−0.0689 (4)	0.09543 (9)	0.0521 (7)	
C18	0.0539 (3)	−0.0329 (5)	0.11120 (12)	0.0716 (9)	
H18	0.0539	0.0402	0.1405	0.086*	
C19	−0.0598 (3)	−0.1044 (7)	0.08383 (17)	0.0976 (12)	
H19	−0.1357	−0.0802	0.0950	0.117*	
C20	−0.0607 (5)	−0.2105 (7)	0.04053 (17)	0.1034 (14)	
H20	−0.1371	−0.2595	0.0224	0.124*	
C21	0.0496 (5)	−0.2446 (5)	0.02384 (13)	0.0921 (12)	
H21	0.0482	−0.3150	−0.0060	0.111*	
C22	0.1643 (3)	−0.1750 (4)	0.05112 (11)	0.0680 (8)	
H22	0.2398	−0.1996	0.0397	0.082*	

### Atomic displacement parameters (Å^2^)

**Table:**

	*U*^11^	*U*^22^	*U*^33^	*U*^12^	*U*^13^	*U*^23^
F1	0.1048 (16)	0.1189 (18)	0.1105 (16)	0.0546 (14)	0.0040 (13)	0.0346 (14)
S1	0.1037 (6)	0.0484 (4)	0.0428 (4)	−0.0095 (4)	0.0210 (4)	0.0047 (3)
O1	0.0912 (15)	0.0817 (15)	0.0405 (10)	0.0164 (12)	−0.0058 (10)	−0.0154 (10)
N1	0.0710 (14)	0.0460 (12)	0.0316 (10)	0.0060 (11)	0.0050 (10)	−0.0024 (9)
N2	0.0605 (13)	0.0358 (11)	0.0379 (11)	0.0003 (10)	0.0055 (10)	0.0029 (8)
C1	0.0627 (18)	0.0552 (17)	0.0482 (15)	0.0041 (15)	−0.0013 (13)	0.0088 (13)
C2	0.075 (2)	0.063 (2)	0.0670 (19)	0.0095 (17)	0.0033 (16)	0.0058 (15)
C3	0.064 (2)	0.087 (2)	0.072 (2)	0.0230 (18)	0.0127 (17)	0.0279 (19)
C4	0.064 (2)	0.121 (3)	0.0510 (18)	0.020 (2)	−0.0025 (15)	0.020 (2)
C5	0.0630 (18)	0.099 (3)	0.0424 (15)	0.0080 (19)	0.0008 (14)	0.0019 (15)
C6	0.0500 (15)	0.0624 (17)	0.0360 (13)	−0.0023 (14)	0.0080 (11)	0.0092 (12)
C7	0.0567 (16)	0.0580 (17)	0.0350 (13)	−0.0042 (14)	0.0027 (12)	−0.0001 (12)
C8	0.0644 (17)	0.0389 (14)	0.0309 (12)	−0.0067 (13)	−0.0007 (12)	−0.0060 (10)
C9	0.0685 (18)	0.0413 (15)	0.0468 (14)	0.0062 (14)	0.0038 (13)	0.0037 (11)
C10	0.0657 (17)	0.0346 (13)	0.0452 (14)	−0.0024 (13)	0.0125 (13)	0.0041 (11)
C11	0.072 (2)	0.0539 (18)	0.0614 (17)	−0.0025 (15)	0.0122 (15)	−0.0097 (14)
C12	0.101 (3)	0.065 (2)	0.0594 (19)	−0.012 (2)	0.0029 (18)	−0.0140 (16)
C13	0.143 (3)	0.0475 (19)	0.0521 (18)	−0.005 (2)	0.029 (2)	−0.0051 (14)
C14	0.118 (3)	0.0539 (19)	0.074 (2)	0.018 (2)	0.051 (2)	0.0056 (16)
C15	0.076 (2)	0.0505 (17)	0.0659 (18)	0.0077 (16)	0.0252 (16)	0.0086 (14)
C16	0.0652 (17)	0.0459 (15)	0.0415 (13)	0.0018 (13)	0.0126 (12)	0.0053 (11)
C17	0.0682 (18)	0.0428 (15)	0.0455 (14)	−0.0060 (14)	0.0114 (13)	0.0071 (12)
C18	0.074 (2)	0.078 (2)	0.0655 (19)	−0.0069 (18)	0.0196 (17)	0.0071 (16)
C19	0.067 (2)	0.127 (4)	0.098 (3)	−0.021 (2)	0.014 (2)	0.022 (3)
C20	0.100 (3)	0.116 (3)	0.083 (3)	−0.051 (3)	−0.011 (2)	0.022 (2)
C21	0.135 (4)	0.074 (2)	0.060 (2)	−0.038 (3)	0.001 (2)	−0.0021 (17)
C22	0.092 (2)	0.0541 (17)	0.0564 (17)	−0.0092 (17)	0.0109 (16)	−0.0003 (14)

### Geometric parameters (Å, °)

**Table:**

F1—C3	1.360 (4)	C10—C11	1.376 (4)
S1—C8	1.678 (3)	C11—C12	1.375 (4)
O1—C7	1.213 (3)	C11—H11	0.9300
N1—C7	1.385 (3)	C12—C13	1.365 (5)
N1—C8	1.401 (3)	C12—H12	0.9300
N1—H1	0.8600	C13—C14	1.367 (5)
N2—C8	1.323 (3)	C13—H13	0.9300
N2—C9	1.467 (3)	C14—C15	1.391 (4)
N2—C16	1.475 (3)	C14—H14	0.9300
C1—C2	1.378 (4)	C15—H15	0.9300
C1—C6	1.383 (4)	C16—C17	1.508 (4)
C1—H1A	0.9300	C16—H16A	0.9700
C2—C3	1.361 (4)	C16—H16B	0.9700
C2—H2	0.9300	C17—C18	1.372 (4)
C3—C4	1.360 (5)	C17—C22	1.385 (4)
C4—C5	1.376 (4)	C18—C19	1.383 (5)
C4—H4	0.9300	C18—H18	0.9300
C5—C6	1.389 (4)	C19—C20	1.364 (6)
C5—H5	0.9300	C19—H19	0.9300
C6—C7	1.482 (4)	C20—C21	1.356 (6)
C9—C10	1.507 (3)	C20—H20	0.9300
C9—H9A	0.9700	C21—C22	1.386 (5)
C9—H9B	0.9700	C21—H21	0.9300
C10—C15	1.375 (4)	C22—H22	0.9300
			
C7—N1—C8	122.6 (2)	C12—C11—C10	121.2 (3)
C7—N1—H1	118.7	C12—C11—H11	119.4
C8—N1—H1	118.7	C10—C11—H11	119.4
C8—N2—C9	122.0 (2)	C13—C12—C11	120.2 (3)
C8—N2—C16	123.8 (2)	C13—C12—H12	119.9
C9—N2—C16	113.9 (2)	C11—C12—H12	119.9
C2—C1—C6	121.1 (3)	C12—C13—C14	119.6 (3)
C2—C1—H1A	119.4	C12—C13—H13	120.2
C6—C1—H1A	119.4	C14—C13—H13	120.2
C3—C2—C1	118.2 (3)	C13—C14—C15	120.2 (3)
C3—C2—H2	120.9	C13—C14—H14	119.9
C1—C2—H2	120.9	C15—C14—H14	119.9
C4—C3—F1	118.4 (3)	C10—C15—C14	120.4 (3)
C4—C3—C2	123.0 (3)	C10—C15—H15	119.8
F1—C3—C2	118.5 (3)	C14—C15—H15	119.8
C3—C4—C5	118.3 (3)	N2—C16—C17	110.32 (19)
C3—C4—H4	120.8	N2—C16—H16A	109.6
C5—C4—H4	120.8	C17—C16—H16A	109.6
C4—C5—C6	120.9 (3)	N2—C16—H16B	109.6
C4—C5—H5	119.5	C17—C16—H16B	109.6
C6—C5—H5	119.5	H16A—C16—H16B	108.1
C1—C6—C5	118.4 (3)	C18—C17—C22	118.8 (3)
C1—C6—C7	123.7 (2)	C18—C17—C16	121.1 (3)
C5—C6—C7	117.8 (3)	C22—C17—C16	120.1 (3)
O1—C7—N1	121.1 (2)	C17—C18—C19	120.5 (3)
O1—C7—C6	122.2 (2)	C17—C18—H18	119.7
N1—C7—C6	116.7 (2)	C19—C18—H18	119.7
N2—C8—N1	116.7 (2)	C20—C19—C18	120.1 (4)
N2—C8—S1	124.9 (2)	C20—C19—H19	120.0
N1—C8—S1	118.3 (2)	C18—C19—H19	120.0
N2—C9—C10	112.4 (2)	C21—C20—C19	120.2 (4)
N2—C9—H9A	109.1	C21—C20—H20	119.9
C10—C9—H9A	109.1	C19—C20—H20	119.9
N2—C9—H9B	109.1	C20—C21—C22	120.3 (4)
C10—C9—H9B	109.1	C20—C21—H21	119.8
H9A—C9—H9B	107.8	C22—C21—H21	119.8
C15—C10—C11	118.4 (3)	C17—C22—C21	120.0 (3)
C15—C10—C9	120.1 (3)	C17—C22—H22	120.0
C11—C10—C9	121.5 (3)	C21—C22—H22	120.0

### Hydrogen-bond geometry (Å, °)

**Table:**

*D*—H···*A*	*D*—H	H···*A*	*D*···*A*	*D*—H···*A*
C9—H9B···S1	0.97	2.55	3.076 (3)	114
N1—H1···S1^i^	0.86	2.61	3.422 (2)	159
C1—H1A···S1^i^	0.93	2.87	3.727 (3)	154
C4—H4···O1^ii^	0.93	2.50	3.322 (4)	147

Symmetry codes: (i) −*x*+1, −*y*, −*z*; (ii) −*x*+3/2, *y*−1/2, −*z*+1/2.
